# Modelling of the ICF core sets for chronic ischemic heart disease using the LASSO model in Chinese patients

**DOI:** 10.1186/s12955-018-0957-0

**Published:** 2018-07-11

**Authors:** Meng You, Wen Fang, Xu Wang, Tiantong Yang

**Affiliations:** 1 Collaborative Innovation Center of Judicial Civilization, Key Laboratory of Evidence Science, China University of Political Science and Law, Ministry of Education, 25 Xitucheng Road, Beijing, 100040 China; 2 Beijing Jiaotong University, Beijing, China

**Keywords:** International classification of functioning, disability, and health, Least absolute shrinkage and selection operator model, Chronic ischemic heart disease, Functioning

## Abstract

**Background:**

This study aimed to examine the associations among the International Classification of Functioning, Disability, and Health (ICF) core sets relevant to chronic ischemic heart disease (CIHD) using the least absolute shrinkage and selection operator (LASSO) model based on the ICF core sets scale in Chinese patients.

**Methods:**

This was a prospective study of 120 patients with CIHD selected from January 2013 to June 2014 at the Fada Institute of Forensic Medicine & Science (Beijing, China). Functioning was qualified using the ICF core sets checklist for CIHD (Chinese version). The variables of core set categories of the ICF assessment scale for CIHD were entered into the LASSO model for mining dependencies among those variables. Graphical modeling was applied using LASSO generalized linear models.

**Results:**

“Muscle endurance functions”, “sensations associated with cardiovascular and respiratory functions”, “blood vessel functions”, and “heart functions” were the most injured in CIHD status. “Recreation and leisure” and “intimate relationships” were the most affected in CIHD status. “General social support services, systems, and policies” and “acquaintances, peers, colleagues, neighbors, and community members” were important for the outcome of functional status of the CIHD patient. “Economic self-sufficiency” and “family relationships” of the CIHD patient were not undermined in most cases.

**Conclusions:**

Graphical modeling can be used to describe associations between different areas of functioning in CIHD patients. The results suggest that these associations could be used as basis to improve rehabilitation and provide a deeper understanding of functioning in Chinese CIHD patients.

**Electronic supplementary material:**

The online version of this article (10.1186/s12955-018-0957-0) contains supplementary material, which is available to authorized users.

## Background

Chronic ischemic heart diseases (CIHD) encompass stable angina pectoris (or symptoms felt to be related to coronary artery disease (CAD), such as dyspnea), symptomatic CAD that became asymptomatic with appropriate treatments and regular follow-up, and newly symptomatic CAD deemed to already be in a chronic and stable condition [1]. Therefore, CIHD include heart conditions that exclude situations in which coronary artery thrombosis acutely dominates presentation [1]. CIHD is characterized by episodes of unbalanced oxygen need/supply, leading to transient ischemia/hypoxia and symptoms of angina [2–4]. The prevalence of CIHD is 4–7% in men aged 45–64 years and 12–14% in men aged 65–84 years, compared to 5.7% in women aged 45–64 years and 10–12% in women aged 65–84 years [5].

The estimated cardiac mortality in populations of patients with CIHD is 1.2–2.4% per year [6–8], but there is a wide variability among patients because of comorbidities such as hypertension and diabetes mellitus [1]. Assessing the prognosis is important because patients with a good prognosis could avoid unnecessary invasive tests and revascularization procedures [1].

The International Classification of Functioning, Disability, and Health (ICF) is an international classification developed by the World Health Organization (WHO) [9, 10]. In contrast to other international classifications, the ICF highlights the structure and measurement of “health components”, and emphasizes on “functioning” rather than “impairment” to provide information on abnormal health status [11, 12]. In order to ensure the wide application of ICF in clinical practice and studies measuring the health status of diseases, the WHO has developed the corresponding ICF core sets for evaluating the health status of some diseases, which refers to the selection of the fewest ICF categories relevant to the patient’s function, disability, and health based on specific diseases and environments [13–15]. These core sets are a secure, fast, and accurate instrument for the application of ICF in clinical practice [16]. The ICF aims at providing a unified language for the description of health conditions in rehabilitation [17, 18]. Based on the ICF as a common language, it is possible to analyze functioning beyond the study of the incidence and prevalence of health conditions and beyond the limited aspects of functioning such as activities of daily living [19, 20].

With the ICF, it is possible to analyze the relationship of elements of functioning on the level of single categories. A promising approach to describe the complex relationships in human functioning is graphical modeling. The least absolute shrinkage and selection operator (LASSO) graphical model is a comprehensive probabilistic tool to analyze and visualize dependencies between random variables. The LASSO graphical model constricts a regression coefficient and thus, directly turns a portion of the coefficients with small absolute value to 0 [21, 22]. The LASSO graphical model has the algorithmic computational complexity equal to that of the least square regression and is effective for selecting variables that have intense effects on dependent variables [23–25]. The correlations among these variables can be directly shown by functional topographic maps. Some authors have used graphical modeling to investigate ICF core sets. For example, Becker et al. [26] used graphical modeling to investigate the associations among functional categories of head and neck cancer in ICF. Kalisch et al. [27] used graphical models to investigate functional data in ICF and they believe that this method can become a tool for functional analysis. Similar to the above studies, Strobl et al. [28] used graphical modeling to study ICF and demonstrated “paths” related to “structures”, which was a good illustration for the associations among ICF categories. Ehrmann et al. [29] used graphical modeling to describe and understand the functioning of people living with a health condition.

Nevertheless, no core sets specific to Chinese patients with CIHD are available. Since the ICF is an international and authoritative tool for functioning evaluation, the present study aimed to examine the associations among the ICF core sets relevant to CIHD using the LASSO model based on the ICF core sets scale in Chinese patients. An ICF theoretical structure-based CIHD functional topographic map was then created to provide evidence for CIHD-related impairment evaluation and give a deeper understanding of functioning in CIHD patients.

## Methods

### Study design

This was a prospective study of 120 patients with CIHD selected from January 2013 to June 2014 at the Fada Institute of Forensic Medicine & Science (Beijing, China). The Fada Institute of Forensic Medicine & Sciences is affiliated to the China University of Political Science and Law. It is also the national-level forensic expertise agency of China. In order to meet the needs of judicial trials and being entrusted by the court or insurance companies, this institution has to perform functional assessment for more than 3000 persons each year. Therefore, researchers in this institution should have the ability to perform objective and comprehensive assessment for functions of the human body. Patient data were reviewed by an accreditation agency, which fully disclosed medical condition of the patients to meet the requirements of comprehensiveness and sufficiency. Sample data involved in this study were all consecutive subjects that were cross-examined at the court. Medical records meeting the CIHD diagnostic criteria were included in the study.

In China, there is a lack of data for the misdiagnosis rate of chronic ischemic heart disease, but there are data about the misdiagnosis rate of acute myocardial infarction (AMI) (21.962% from 2004 to 2013). Since chronic ischemic heart disease and AMI have common disease outcomes, we used the AMI misdiagnosis rate as estimation. Based on the formula:

$$ n=\frac{u_{\alpha}^2{\sigma}^2}{\delta^2} $$

Power was set at 0.8 and β at 0.2. u was the Z score and α (two-sided) was set at 0.05.

The overall misdiagnosis rate was 21.962% × 0.2 = 4.3924%. *σ* was set at 21.962 and δ at 4. The sample size was determined as 116 and a total of 120 subjects were included for the present analysis.

This study was approved by the ethics committee of the China Collaborative Innovation Center of Judicial Civilization (Beijing). Informed consent was obtained from all participants included in the study.

### Subjects

The inclusion criteria were: 1) consistent with the diagnosis of angina (I20) or CIHD (I25) according to the International Classification of Diseases-10th Revision (ICD-10); 2) age ≥ 18 years; and 3) no recent movement disorders or difficulties caused by surgeries, trauma, etc. Patients with difficulties in movement from recent surgery, trauma, or any other causes were excluded.

### Data acquisition

Data in this study were all from lawsuits cases of personal injury claims or cases qualified with health insurance, including all medical records of patients that were approved by cross-examination at the court. These data can reflect the health conditions of the patients comprehensively. In this study, 16 experts were invited to evaluate qualifier scale of relevant categories based on medical data of patients and ICF CIHD checklist (Chinese version). If there were cases with unclear medical records, then data were supplemented by interview or professional observation.

The 16 clinical specialists were responsible for data collection; 12 were cardiovascular specialists and four were rehabilitation specialists. They received special training. They collected data from the 120 patients with CIHD using the ICF Core Sets checklist for CIHD (Chinese language version) [30]. In this checklist, a total of 46 categories are included under the four first categories (b. body functions, s. body structures, d. activities and participation, and e. environmental factors; Table 1). For body functions and structures, the level of impairment was assessed according to the ICF qualifier scale: 0, no impairment; 1, mild impairment; 2, moderate impairment; 3, severe impairment; and 4, complete impairment. For the environmental factor component: 0, no barriers; 1, mild barriers; 2, moderate barriers; 3, severe barriers; and 4, complete barriers. For activities and participation: 0, no difficulty; 1, mild difficulty; 2, moderate difficulty; 3, severe difficulty; and 4, complete difficulty. For all categories, not specified was encoded as 8, and not applicable was encoded as 9.

**Table 1:** Short description of the ICF categories used for the graphs

ICF code	Category description	ICF code	Category description	ICF code	Category description	ICF code	Category description
	*Chapter: structures of the cardiovascular, immunological, and respiratory systems*		*Chapter: mental functions*		*Chapter: general tasks and demands*		*Chapter: products and technology*
s410	Structure of cardiovascular system	b130	Energy and drive functions	d230	Carrying out daily routine	e110	Products or substances for personal consumption
s430	Structure of respiratory system	b134	Sleep functions	d240	Handling stress and other psychological demands	e125	Products and technology for communication
		b144	Memory functions		*Chapter: mobility*		
		b152	Emotional functions	d430	Lifting and carrying objects	e155	Design, construction and building products and technology of buildings for private use
			*Chapter: functions of the cardiovascular, hematological, immunological and respiratory systems*	d450	Walking		*Chapter: natural environment and human-made changes to environment*
		b410	Heart functions	d455	Moving around	e225	Climate
		b415	Blood vessel functions	d460	Moving around in different locations	e250	Sound
		b420	Blood pressure functions		*Chapter: self-care*	e260	Air quality
		b455	Exercise tolerance functions	d570	Looking after one’s health		*Chapter: support and relationships*
		b460	Sensations associated with cardiovascular and respiratory functions		*Chapter: Domestic life*	e310	Immediate family
			*Chapter: functions of the digestive, metabolic and endocrine systems*	d620	Acquisition of goods and services	e315	Extended family
		b530	Weight maintenance functions	d630	Preparing meals	e320	Friends
			*Chapter: neuromusculoskeletal and movement-related functions*	d640	Doing housework	e325	Acquaintances, peers, colleagues, neighbors, and community members
		b730	Muscle power functions		*Chapter: interpersonal interactions and relationships*	e330	People in positions of authority
		b740	Muscle endurance functions	d760	Family relationships	e355	Health professionals
				d770	Intimate relationships		*Chapter: attitudes*
					*Chapter: major life areas*	e410	Individual attitudes of immediate family members
				d850	Remunerative employment	e450	Individual attitudes of health professionals
				d870	Economic self-sufficiency		*Chapter: services, systems and policies*
					*Chapter: community, social and civic life*	e570	Social security services, systems and policies
				d920	Recreation and leisure	e575	General social support services, systems and policies
						e580	Health services, systems and policies

### Data processing and interpretation

The LASSO model was used to analyze the conditional dependence among the categorical variables of core sets for CIHD using MATLAB 8.3 (MathWorks, Natick, MA, USA) [26, 28]. In the present model, there were 120 subjects and 46 ICF variables. The minimum mean square error (MMSE) and the coefficient vector were obtained by LASSO estimation; they were both considered as dependence coefficients.

The dependence coefficient showed the relative correlations among different categories. Analogical reasoning was applied to other variables to induce their corresponding variables.

## Results

### Characteristics of the patients

Among the 120 patients, there were 85 males and 35 females, aged 35–82 years. Based on the ICD-10, 79 cases were confirmed with angina (I20) and 41 with CIHD (I25). According to the New York Heart Association Functional Classification [31], 52 patients were class I, 37 were class II, 27 were class III, and four were class IV.

### Confinement degrees of the categories

**Fig. 1:**
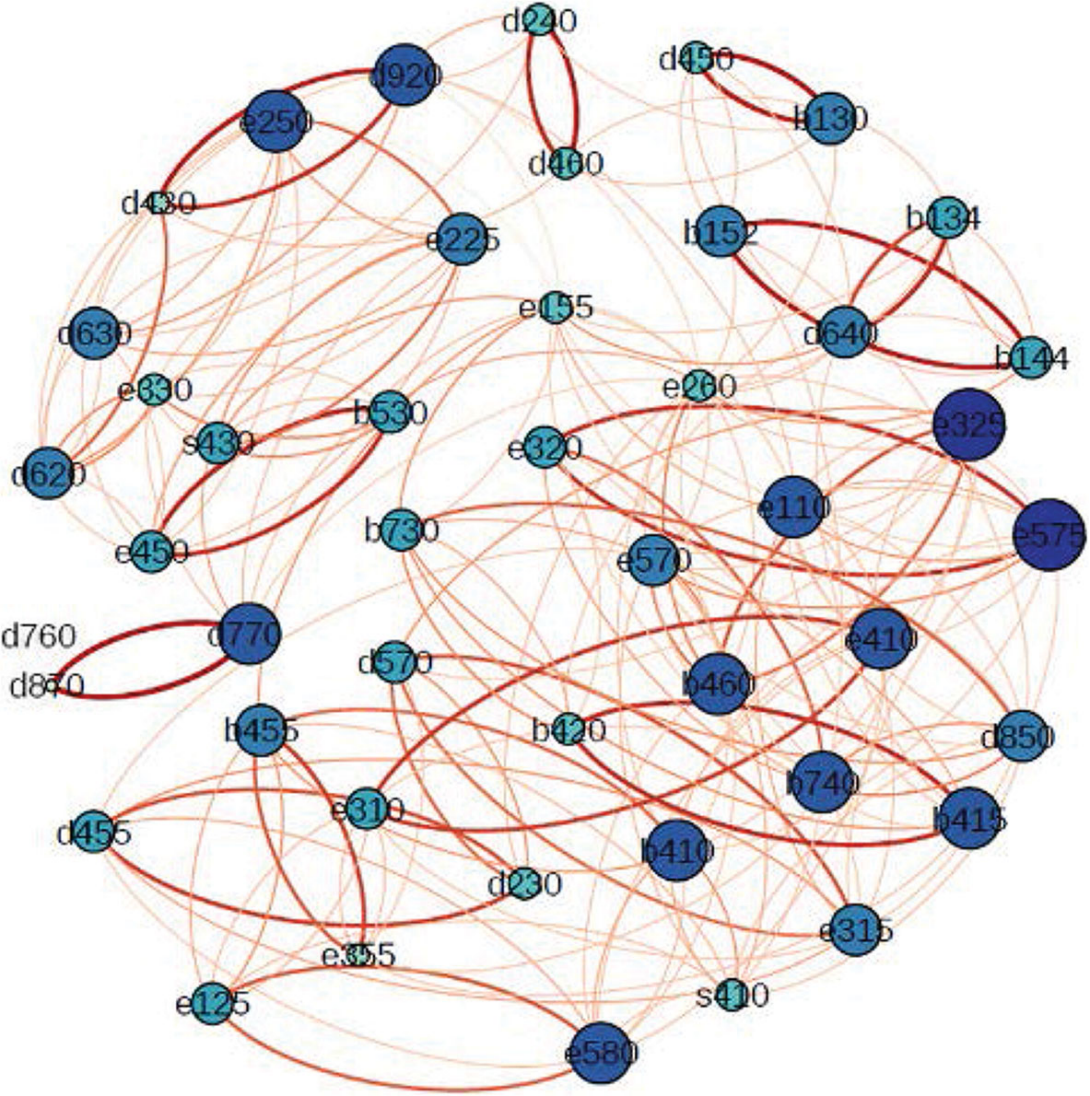
Interaction diagram of classification of ICF core set related to CIHD. Thicker links indicate more heavy a node weigh in relations to others

Individual scores are presented in the Additional file 1: Table S1. In this study, “out degree” stands for the frequency of appearance of a variable compared to others. The larger the frequency is, the more impact a variable has on other variables [32]. A direct weighted network diagram based on the dependence coefficients of different categories was plotted, and the relevant characteristics were analyzed. The maximum out degree of a node/category was 7, while the minimum was 2. The thicker the link was, the more heavily a node was weighted compared to others (Fig. 1). The out degrees of different categories are shown in Table 2. In the category of “body structures” (s), the out degree of s410 was 4, indicating that the “structures of the cardiovascular system” were involved in CIHD status. In the category of “body functions” (b), the out degrees of b740, b460, b415, and b410 were 6, indicating that the “muscle endurance functions”, “sensations associated with cardiovascular and respiratory functions”, “blood vessel functions”, and “heart functions” were the most injured in CIHD status. In the category of “activities and participation” (d), the out degrees of d920 and d770 were 6, indicating that “recreation and leisure” and “intimate relationships” were the most affected in CIHD status. In the category of “environmental factors” (e), the out degrees of e575 and e325 were 7, indicating that “general social support services, systems, and policies” and “acquaintances, peers, colleagues, neighbors, and community members” were important for the outcome of functional status of the CIHD patient. Among all categories, the out degrees of d870 and d760 were the smallest, indicating that “economic self-sufficiency” and “family relationships” of the CIHD patient were not undermined in most cases. These results reveal that the main direction for CIHD status evaluation should be first determined, with major emphasis given to the category with the highest confinement degree.

**Table 2:** Out degree of classification core set node related to CIHD

ICF code	Out degree	ICF code	Out degree	ICF code	Out degree	ICF code	Out degree
s410	4	b740	6	d920	6	e575	7
s430	3	b460	6	d770	6	e325	7
		b415	6	d850	5	e580	6
		b410	6	d640	5	e410	6
		b455	5	d630	5	e250	6
		b152	5	d620	5	e110	6
		b130	5	d570	4	e570	5
		b730	4	d455	4	e315	5
		b530	4	d460	3	e225	5
		b144	4	d450	3	e450	4
		b134	4	d240	3	e320	4
		b420	3	d230	3	e310	4
				d430	2	e125	4
				d870	1	e330	3
				d760	0	e260	3
						e155	3
						e355	2

### Relationship between two categories

A total of 2116 dependence coefficients were acquired during the evaluation of the different categories of core sets for CIHD using ICF, including 14 dependence coefficients > 0.75 and 16 dependence coefficients < 0.25. The graphical model showed several complete bidirectional feedback loops in the core sets of CIHD categories based on different dependence coefficients (Tables 3 and 4).

**Table 3:** Dependence coefficient of classification related to CIHD core set (dependence coefficient ≥ 0.75)

Category	d770	d870	b530	e450	d920	d240	d460	b130	d450	b152	b144	d640	b415	b420
b130	0	0	0	0	0	0.002	0.072	0	0.858	0	0	0	0	0
b134	0	0	0	0	0	0	0	0	0	0	0.081	0.779	0	0
b144	0	0	0	0	0	0	0	0	0	0.862	0	0.11	0	0
b152	0	0	0	0	0	0	0	0	0.024	0	0.857	0	0	0
b415	0	0	0	0	0	0	0	0	0	0	0	0.026	0	0.758
b420	0	0	0	0	0	0	0	0	0	0	0	0.015	0.773	0
b530	0	0	0	0.755	0	0	0	0	0	0	0	0	0	0
d240	0	0	0	0	0.056	0	0.869	0	0	0	0	0	0	0
d430	0	0	0	0	0.86	0	0	0	0	0	0	0	0	0
d450	0	0	0	0	0	0	0	0.916	0	0.09	0	0.017	0	0
d460	0	0	0	0	0	0.778	0	0.03	0	0	0	0	0	0
d770	0	0.968	0	0	0	0	0	0	0	0	0	0	0	0
d870	0.968	0	0	0	0	0	0	0	0	0	0	0	0	0
e450	0	0	0.816	0	0	0	0	0	0	0	0	0	0	0

**Table 4:** Dependence coefficient of classification related to CIHD core set (dependence coefficient ≤ 0.25)

Category	s430	e330	b620	e250	d630	b730	b740	e570	e260	b460	e225	d570	b410	e155	e110	s410
s410	0	0	0	0	0	0	0	0.128	0	0	0	0	0.150	0	0	0
b134	0	0	0	0	0	0	0	0	0.041	0	0	0	0	0.046	0	0
b144	0	0	0	0	0	0	0	0	0.231	0	0	0	0	0	0	0
b152	0	0	0	0	0	0	0	0	0.005	0	0	0	0	0	0	0.020
b740	0	0	0	0	0	0	0	0.011	−0.040	0	0	0	0	−0.021	0	0
d240	0	0	0	0	0.094	0	0	0	0	0	0	0	0	0	0	0
d450	0	0	0	0	0	0	0	0	0	0	0	0	0	0	0	0
d455	0	0	0	0	0	0	0	0.048	0	0	0	0	0	0	0	0
d460	0	0	0	0	0	0	0	0	0	0	0	0	0	0	0	0
d570	0	0	0	0	0	0	0	0	0	0	0	0	0	0	0	0
d630	0	0	0.058	0.047	0	0	0	0	0	0	0.042	0	0	0	0	0
d760	0	0	0	0	0	0	0	0	0	0	0	0	0	0	0	0
d770	0.208	0.046	0	0	0	0	0	0	−0.052	0	0.067	0	0	0.078	0	0
d870	0	0	0	0	0	0	0	0	0	0	0	0	0	0	0	0
d920	0	0	0	0	0.235	0	0	0	−0.095	0	0	0	0	− 0.197	0.045	0
e110	0	0	0	0	0	0	0	0	0	0	0	0	0	0.141	0	0
e125	0	0	0	0	0	0	0	0	0	0	0	0	0	0	0	0.064
e155	0	0	0	0	0	0.021	0	0	0.014	0	0	0	0	0	0.012	0
e260	0	0	0	0	0	0	0	0.027	0	0.029	0	0	0	0	0	0
e310	0	0	0	0	0	0	0.014	0	0	0	0	0	0	0	0	0
e355	0	0	0	0	0	0	0	0	0	0	0	0	0	0	0	0
e450	0	0	0.016	0	0.021	0	0	0	0	0	0.240	0	0	0	0	0
e580	0	0	0	0	0	0	0	0	0	0.073	0	0	0.206	0	0	0.010

Among all categories with dependence coefficients > 0.75 (Table 3), d770 and d870 had the largest dependence coefficient (0.968), indicating that “intimate relationships” was closely correlated to “economic self-sufficiency”; the dependence coefficient for b130 and d450 was 0.916, indicating that “energy and drive functions” was closely correlated with “walking”; d460 and d240, b152 and b144, and d920 and d430 had dependence coefficients of 0.869, 0.862, and 0.86, respectively, indicating strong correlations between these categories in CIHD status, and the confinement of one category affects the other. Among the categories with dependence coefficient < 0.25 (Table 4), e155 and d920 had dependence coefficients of − 0.197, showing a negative correlation, indicating that “recreation and leisure” was not confined but enhanced although “design, construction, and building products and technology of buildings for private use” was confined; the dependence coefficients between e260 and d920/d770 were − 0.095 and − 0.052, indicating that “recreation and leisure” and “intimate relationships” were enhanced even though “air quality” was confined. The categories showing either positive or negative correlations indicated that the correlations among different confined categories should be studied when evaluating CIHD-related status so as to determine the interactions among different categories and comprehensively evaluate the confined functioning status.

### Correlations among multiple categories

Correlations existed among multiple categories of ICF core sets for CIHD. The dependence coefficients for b134, d640, d450, b152, and b144 linked to b134 were 0.779, 0.059, 0.090, 0.857, and 0.228, respectively; if the above category order was reversed, the dependence coefficients were 0.857, 0.862, 0.024, 0.017, and 0.652, respectively, indicating that “sleep functions” was correlated with “doing housework”, “walking”, “emotional function”, and “memory functions” and thus, a complete bidirectional feedback loops existed. This also suggested that the aforementioned categories can be confined in CIHD status, and the confinement degree was affected by other categories of the same feedback loop, indicating that a holistic approach is required for evaluating CIHD status, and to clarify the essential pathophysiological mechanisms and prognostic rules of functioning confinement for the CIHD patient with respect to correlation among multiple confined components.

## Discussion

LASSO is a method of compressive estimation proposed by Tibshirani in 1996 and is suitable for managing multicollinearity problems [33]. It is a regression method that can simultaneously perform parameter estimation and implement variable selection. Compared to other models, a graphical model can represent the dependence of the probability of variables using graphics, as well as represent joint probability distribution of variables associated with the model. It provides a simple way to visualize the structure of probabilistic model. By observing the graph, readers can understand the nature of the model more deeply. Induction of advanced model and complex calculations in the machine learning process can be expressed based on graphical calculation. The graphs implicitly carry the underlying mathematical expressions. Some authors have used graphical modeling to investigate ICF core sets [26–29]. The associations and degree of association were intuitively displayed by graphs, which can be a reference for diagnosis, functional limitation, functional assessment, comprehensive treatment and rehabilitation involved in chronic ischemic heart disease.

As an international standard for describing function and health, the ICF supplements abundant health status information on individuals’ and population’s “functioning”, and demonstrates different functioning levels between disease occurrence and lesion outcome [34]. ICF is increasingly used for the evaluation of disease and lesion outcome in clinical practice and research [35]. This study aimed to examine the associations among the ICF core sets relevant to CIHD using the LASSO model based on the ICF core sets scale in Chinese patients. The results showed that “muscle endurance functions”, “sensations associated with cardiovascular and respiratory functions”, “blood vessel functions”, and “heart functions” were the most injured in CIHD. “Recreation and leisure” and “intimate relationships” were the most affected in CIHD status. “General social support services, systems, and policies” and “acquaintances, peers, colleagues, neighbors, and community members” were important for the outcome of functional status of the CIHD patient. “Economic self-sufficiency” and “family relationships” of the CIHD patient were not undermined in most cases. Therefore, graphical modeling can be used to describe associations between different areas of functioning in CIHD patients. The results suggest that these associations could be used as basis to improve rehabilitation and provide a deeper understanding of functioning in Chinese CIHD patients.

ICF is a new classification system for health components based on interaction patterns. Individual functions of specific fields result from the interactions and complex correlations between health status and background factors (including environmental and individual factors). The “function”, “health”, and “disability” are independent and correlated, and they integrate health status, function, impairment, and background factors into a bidirectional interactive unified system [36, 37]. Therefore, ICF is a commonly used instrument that provides a unified and standard system for the description of health and health-related status worldwide [38]. Using the LASSO algorithm for the mathematical modeling of sample data and drawing a topographic diagram of correlations among ICF core sets for CIHD helps analyze the confinement degrees among body structures, functions, and activity participation, as well as their interactions in CIHD. It also provides robust scientific evidence for the introduction of ICF in functional recovery and disability evaluation, and improvement of the influence and recognition of evaluation results.

A previous study in patients with head and neck cancer showed that graphical modeling could be used to describe the associations among areas of functioning in these patients, providing a better basis for the management of these patients [26]. Another study in patients with traumatic brain injury (TBI) showed that the LASSO model could be used to construct high-order functional networks for application in clinical settings [22]. Nevertheless, compared with the former ICF core classification sets of TBI [22], the number of classifications was higher in the present study and their complexity was also higher. For the first time, a negative correlation was underlined.

In the present study, “muscle endurance functions”, “sensations associated with cardiovascular and respiratory functions”, “blood vessel functions”, and “heart functions” were the most injured in CIHD. “Recreation and leisure” and “intimate relationships” were the most affected in CIHD. These results are in line with previous studies of ICF in patients with heart diseases [30, 39, 40] and consistent with the course of the disease. “General social support services, systems, and policies” and “acquaintances, peers, colleagues, neighbors, and community members” are important for the outcome of functional status of the CIHD patient, as supported by a number of studies [41–43].

The results of the limited extent of category suggest that during CIHD-related disability assessment, the direction of the assessment should be clarified, focusing on the evaluation of the restricted categories that are more likely to be affected according to the out-degree of different classifications, and reasonable arrangement of the assessment sequence. Since there are positive and negative correlations among the classifications, the assessment should focus on the connection between two restricted categories to determine the degree of mutual influence among various categories, thus comprehensively and accurately evaluate the status of limited function of patients with CIHD during the evaluation of their disability.

The associations among multiple categories imply that there is a restricted situation in the above-mentioned classifications in the CIHD state, and the degree of restriction is related to the other categories in the feedback loop. This suggests that we should have the holistic concept in disability assessment, and recognize the fundamental pathophysiological process and outcome of the limited function of CIHD from the perspective of interdependencies of multiple restricted components.

The limitations of the present study are that the score data obtained by the interviews were subjective. In addition, it is still difficult to introduce the ICF standard in the current Chinese health care system. The LASSO approach itself has some limitations such as the consideration of only a small number of possible graphs [44], also as the dynamic changes in the relations among factors [45, 46]. Finally, 16 specialists visited the patients, which could introduce some bias. Additional studies are still necessary to refine the model.

## Conclusions

In conclusion, graphical modeling can be used to describe associations between different areas of functioning in CIHD patients. The results suggest that these associations could be used as basis to improve rehabilitation and provide a deeper understanding of functioning in Chinese CIHD patients.

## Additional file

Additional file 1: **Table S1.** Scores for each individual item of the ICF core sets. (DOCX 14 kb)

## Abbreviations

CAD: Coronary artery disease
CIHD: Chronic ischemic heart disease
ICF: International Classification of Functioning, Disability, and Health
LASSO: Least absolute shrinkage and selection operator
MMSE: Minimum mean square error
TBI: Traumatic brain injury
WHO: World Health Organization
