# Supplementary material for: Modelling of the ICF core sets for chronic ischemic heart disease using the LASSO model in Chinese patients
Source: Health Qual Life Outcomes. 2018 Jul 11;16:139. doi: 10.1186/s12955-018-0957-0 (PMC6042460; doi:10.1186/s12955-018-0957-0)
Supplement: Supplementary file 1 — Table S1. Scores for each individual item of the ICF core sets. (DOCX 14 kb) [file 12955_2018_957_MOESM1_ESM.docx]

**Table S1.** Scores for each individual item of the ICF core sets.

| Category | Mean ± SD | Category | Mean ± SD | Category | Mean ± SD | Category | Mean ± SD | Category | Mean ± SD |
| --- | --- | --- | --- | --- | --- | --- | --- | --- | --- |
| d760 | 0.01±0.92 | d920 | 0.99±1.53 | b415 | 1.89±1.66 | e325 | 2.45±1.48 | e155 | 2.04±1.12 |
| d770 | 0.20±0.79 | d430 | 0.99±1.52 | b420 | 1.89±1.65 | e225 | 0.45±1.01 | e355 | 2.87±1.13 |
| d870 | 0.20±0.79 | d240 | 1.09±1.57 | b730 | 1.933±1.65 | d570 | 2.58±1.39 | e110 | 2.62±1.18 |
| s430 | 0.39±1.05 | d460 | 1.10±1.58 | d850 | 2.01±1.62 | e315 | 2.56±1.40 | e310 | 2.78±1.09 |
| b530 | 0.53±1.22 | b130 | 1.23±1.62 | b740 | 2.10±1.62 | d230 | 2.60±1.38 | e410 | 2.76±1.11 |
| e450 | 0.55±1.23 | d450 | 1.24±1.62 | e320 | 2.18±1.61 | d455 | 2.59±1.37 | s410 | 3.01±0.96 |
| e330 | 0.58±1.16 | b152 | 1.30±1.64 | e575 | 2.20±1.58 | e580 | 2.70±1.26 |  |  |
| b620 | 0.82±1.42 | b144 | 1.31±1.63 | e570 | 2.32±1.54 | e125 | 2.75±1.23 |  |  |
| e250 | 0.24±0.50 | b134 | 1.38±1.66 | e260 | 1.86±1.41 | b410 | 2.80±1.23 |  |  |
| d630 | 0.65±1.08 | d640 | 1.40±1.66 | b460 | 2.40±1.52 | b455 | 2.82±1.20 |  |  |
